# Development and validation of a recombinant capsid protein-based indirect enzyme-linked immunosorbent assay for serological detection of duck circovirus in commercial flocks

**DOI:** 10.14202/vetworld.2025.3378-3389

**Published:** 2025-11-06

**Authors:** Thnapol Luengyosluechakul, Sittinee Kulprasertsri, Siriluk Jala, Sakuna Phatthanakunanan, Preeda Lertwatcharasarakul

**Affiliations:** 1 Animal Health and Biomedical Sciences Study Program, Faculty of Veterinary Medicine, Kasetsart University, Bangkok 10900, Thailand; 2 Department of Farm Resources and Production Medicine, Faculty of Veterinary Medicine, Kasetsart University, Nakhon Pathom 73140, Thailand; 3 Kamphaeng Sean Veterinary Diagnostic Center, Faculty of Veterinary Medicine, Kasetsart University, Nakhon Pathom 73140, Thailand; 4 Department of Pathology, Faculty of Veterinary Medicine, Kasetsart University, Nakhon Pathom 73140, Thailand

**Keywords:** capsid protein, duck circovirus, indirect enzyme-linked immunosorbent assay, recombinant protein, serodiagnosis

## Abstract

**Background and Aim:**

Duck circovirus (DuCV) is an immunosuppressive pathogen linked to poor growth, feather abnormalities, and increased susceptibility to co-infections, leading to significant economic losses in duck production. Rapid and large-scale serological screening tools are essential for epidemiological surveillance and biosecurity. This study aimed to develop and validate an indirect enzyme-linked immunosorbent assay (iELISA) based on a recombinant capsid (Cap) protein for sensitive and specific detection of antibodies against DuCV.

**Materials and Methods:**

The *cap* gene from a Thai DuCV genotype I isolate was cloned into the pQE-31 vector and expressed in *Escherichia coli* M15. The 27 kDa recombinant Cap protein was purified under denaturing conditions, and its antigenicity was confirmed by Western blotting. The iELISA was optimized by checkerboard titration to determine the optimal antigen coating concentration and serum dilution. Diagnostic sensitivity, specificity, cross-reactivity, repeatability, reproducibility, and agreement with Western blotting were assessed using 80 positive, 103 negative, and 189 field serum samples.

**Results:**

The optimized iELISA used 12 μg/well of antigen and a 1:20 serum dilution, producing the highest positive-to-negative optical density ratio. Receiver operating characteristic curve analysis yielded an area under the curve of 0.996, with 97.5% sensitivity and 98.1% specificity. No cross-reactivity was detected with sera positive for duck Tembusu virus, duck viral enteritis virus, or *Riemerella anatipestifer*. Intra- and inter-assay coefficients of variation were below 6.5% and 9.1%, respectively. Diagnostic agreement with Western blotting across 189 field sera was 91.0%, with a Cohen’s kappa of 0.752, indicating substantial concordance.

**Conclusion:**

The developed recombinant Cap-based iELISA provides a reliable, specific, and reproducible tool for large-scale DuCV serosurveillance. Its high diagnostic accuracy and scalability support its application in flock-level monitoring, pre-movement screening, and epidemiological studies, facilitating improved biosecurity and informed disease control strategies within the duck industry.

## INTRODUCTION

Duck circovirus (DuCV) is a small, non-enveloped, single-stranded DNA virus first identified in Germany in 2003 [1]. Infected ducks typically display abnormal feathering, poor body condition, and low body weight. The virus primarily targets lymphoid organs, leading to immunosuppression and predisposing birds to secondary infections [2–5]. Such pathological effects decrease flock productivity and contribute to substantial economic losses due to the diminished market value of affected ducks [6, 7]. Since its discovery, DuCV has been reported in multiple countries, including the United States, China, South Korea, Hungary, Taiwan, Poland, the United Kingdom, Vietnam, and Thailand [8–16]. In Southeast Asia, particularly Vietnam and Thailand, which are major producers of duck meat [17], DuCV circulation in commercial flocks has been confirmed through molecular diagnostic techniques [13, 15, 18]. These regional findings highlight the need for scalable serological tools to assess flock-level exposure and strengthen disease surveillance and management programs.

The DuCV genome comprises three major open reading frames (ORFs): ORF1, ORF2, and ORF3. ORF1, located on the viral strand, encodes the replication (Rep)-associated protein essential for viral DNA Rep. ORF2, found on the complementary strand, encodes the capsid (Cap) protein, the primary structural and immunogenic component that elicits host immune recognition [19, 20]. ORF3, situated on the complementary strand of ORF1, is associated with apoptosis induction [21, 22]. Based on complete genome sequencing, DuCV is classified into three genotypes, DuCV1, DuCV2, and DuCV3 [1, 23, 24]. Surveillance data from Thailand indicate that circulating strains belong predominantly to genotype I, with no confirmed reports of genotypes II or III [15].

DuCV frequently co-infects with other pathogens, complicating the clinical diagnosis and obscuring its role as a primary etiological agent. This complexity necessitates precise and sensitive diagnostic tools capable of distinguishing DuCV infections from other viral or bacterial diseases of ducks. Conventional polymerase chain reaction (PCR) and real-time PCR assays remain the standard molecular methods, with SYBR Green and TaqMan probe-based formats offering enhanced sensitivity and specificity [11, 25, 26]. In addition, rapid isothermal amplification techniques, such as real-time fluorescence-based recombinase-aided amplification and loop-mediated isothermal amplification (LAMP), have been developed [27, 28]. The LAMP assay targets the DuCV *rep* gene using six primers, enabling rapid, equipment-free detection with visually interpretable results [28]. Recently, recombinase polymerase amplification (RPA) integrated with the clustered regularly interspaced short palindromic repeats/CRISPR-associated protein 12a (CRISPR/Cas12a) and lateral flow strip (LFS) readouts (RPA–CRISPR/Cas12a–LFS) has emerged as an innovative diagnostic platform [29].

Despite increasing reports of DuCV infections worldwide, including recent confirmations of genotype I circulation in Thailand and neighboring Southeast Asian countries [13, 15, 18], there remains a critical lack of validated serological tools for large-scale surveillance. Most available diagnostic approaches, such as conventional and real-time PCR, are designed to detect active viral DNA [11, 25, 26]. While these molecular techniques offer high analytical sensitivity and specificity, they fail to capture the historical or flock-level immune response essential for understanding exposure dynamics and long-term epidemiological patterns. Furthermore, molecular assays require laboratory infrastructure, thermal cycling instruments, and trained personnel, making them less feasible for high-throughput or field-based applications in developing regions [30].

Previous ELISA platforms have demonstrated potential for detecting antibodies against DuCV [19, 20, 31]; however, several technical and contextual limitations persist. First, most reported ELISAs were developed using antigens derived from non-local subgenotypes (e.g., Chinese or Korean isolates), which may not fully represent the antigenic diversity of circulating Thai strains [6, 15]. Sequence variation among DuCV genotypes can significantly influence antigen–antibody binding affinity, leading to reduced diagnostic accuracy when applied to divergent strains [6, 23]. Second, no commercially available ELISA kits for the serological detection of DuCV have been developed to date, and previously reported assays remain limited to in-house use without standardized validation or widespread accessibility. Consequently, there is a pressing need for a standardized, reproducible, and locally adapted serodiagnostic assay for DuCV surveillance that fulfills both analytical and field validation criteria.

This study aimed to develop and validate an indirect ELISA (iELISA) based on a recombinant Cap protein derived from a Thai field isolate of DuCV genotype I for the serological detection of anti-DuCV antibodies. The Cap protein, being the principal structural and immunogenic component of the virus [19, 20], was expressed in *Escherichia coli* and purified under denaturing conditions to ensure epitope preservation and consistent antigenicity. The assay was systematically optimized for antigen concentration and serum dilution, followed by extensive validation of diagnostic sensitivity, specificity, repeatability, reproducibility, and cross-reactivity. Furthermore, diagnostic accuracy was statistically assessed through ROC curve analysis and comparative evaluation with Western blotting using field sera from commercial duck flocks.

By developing a region-specific, recombinant protein-based iELISA with verified performance metrics, this study provides a cost-effective, scalable, and practical serological tool to support large-scale epidemiological surveillance, pre-movement health certification, and biosecurity monitoring in the Southeast Asian duck industry.

## MATERIALS AND METHODS

### Ethical approval

All animal procedures were reviewed and approved by the Institutional Laboratory Animal Care and Use Committee of Kasetsart University, Thailand (approval no. ACKU67-VET-004, 2023). This study was conducted in accordance with the principles and guidelines for the use of animals in research, ensuring animal welfare and ethical standards.

### Study period and location

This study was conducted from November 2023 to November 2024 at the Faculty of Veterinary Medicine, Kasetsart University, Nakhon Pathom, Thailand.

### Sample collection and reference sera preparation

Spleen tissues were collected from clinically affected ducks originating from commercial flocks with suspected DuCV in Thailand and necropsied at the Kamphaeng Saen Veterinary Diagnostic Center, Faculty of Veterinary Medicine, Kasetsart University. Extracted DNA from these tissues was amplified for the *cap* gene by PCR using specific primers and cloned into an expression vector to produce a recombinant Cap protein.

The sample size for diagnostic validation was calculated to achieve 95% confidence intervals (CIs) with a precision of ±5%. The expected diagnostic sensitivity and specificity of 0.95 were assumed based on previous studies, which reported values of 0.95 [19, 20]. The formula is n = Z^2^ × p × (1−p)/d^2^ with Z = 1.96, d = 0.05, and p = 0.95, resulting in approximately 73 samples/group. Our reference panels, comprising 80 positive and 103 negative sera, exceeded this requirement.

In 2023, 80 positive duck sera were collected from 4 farms of layer and meat-type ducks that had clinical symptoms of DuCV infection, such as feather abnormalities, emaciation, and growth retardation, as well as flock-level tissue PCR positive for DuCV. One hundred and three archived negative sera, collected in 2017 from healthy ducks with no prior history of DuCV infection before Thailand’s first report of DuCV in 2022, were used. PCR did not detect DuCV in these sera. Additionally, 189 field serum samples were collected from layer and meat-type ducks aged 2–52 weeks across seven commercial duck farms in central Thailand in 2023 for comparative evaluation of the iELISA and Western blot analysis.

Blood was allowed to clot and then centrifuged to separate the serum, which was aliquoted (about 100 μL) to reduce the number of freeze–thaw cycles. Serum samples were stored at −20°C and tested after a single thaw.

### DNA extraction and PCR analysis

Total DNA was extracted using the Favor Prep Viral DNA/RNA Extraction Kit (Favorgen, Taiwan) according to the manufacturer’s instructions. DuCV infection was confirmed through conventional PCR targeting the *rep* gene using the following specific primers: P1 (5′-CGGCGCTTGTACTCCGTACTC-3′) and P2 (5′-CCCGCGTGGTTTGTAATACTTG-3′), as described by Wang *et al*. [32]. PCR amplification was performed in a reaction volume of 25 μL, comprising Hot Start Master Mix (Apsalagen, Germany), 0.4 μM of each primer, and 100 ng of template. The cycling conditions included an initial denaturation at 95°C for 2 min, followed by 35 cycles of 95°C for 30 s, 58°C for 30 s, and 72°C for 45 s, and a final extension step at 72°C for 5 min. The amplification products were separated by electrophoresis in a 1.5% agarose gel and visualized by GelDoc EZ (Bio-Rad, USA).

### Primer design and gene amplification

Specific primers were meticulously designed based on six conserved nucleotide sequences identified through a comprehensive multiple sequence alignment of Thai field isolates available in the GenBank database (accession numbers: OQ744001-OQ744006) to enhance the amplification of the *cap* gene of DuCV for the expression of recombinant proteins. Sequence alignment was performed using the ClustalW program in BioEdit version 7.2.5 (https://bioedit.software.informer.com/7.2/). The selected primer pair, DuCV_CAPexF2 (5′-GCCGAAGGTACCAAGGCTACGAATCGCAAGAC-3′) and DuCV_CAPexR (5′-GCCGAAGGATCCCCGTTCTATGTCATA CTGCGC-3′), successfully amplified a 676-base pair (bp) fragment corresponding to the *cap* gene. Furthermore, *Bam*HI and *Kpn*I (New England Biolabs, USA) restriction sites were strategically incorporated at the 5′ ends (underlined) of the forward and reverse primers to facilitate directional cloning.

PCR was performed using Phusion High–Fidelity DNA polymerase (Thermo Fisher Scientific, USA) in a 50 μL reaction volume that included 1× high-fidelity buffer, 0.2 mM dNTPs, 0.5 μM of each primer, and 100 ng of DNA template extracted from DuCV-positive spleen tissue. The thermocycling protocol consisted of an initial denaturation step at 98°C for 30 s, followed by 35 cycles of 98°C for 10 s, 60°C for 30 s, and 72°C for 45 s, with a final extension at 72°C for 10 min.

The PCR products were visualized on a 1.5% agarose gel stained with RedSafe nucleic acid staining (Biotium, USA). Bands of the expected size (676 bp) were excised and purified using the FavorPrep Gel/PCR purification mini kit (Favorgen Biotech Corporation, Taiwan) according to the manufacturer’s instructions. The purified DNA fragments were quantified using a NanoDrop 2000 spectrophotometer (Thermo Fisher Scientific) and cloned into the expression vector.

### Cloning and expression of the recombinant Cap protein

The purified 676-bp DuCV *cap* gene was double-digested with *Bam*HI and *Kpn*I restriction enzymes at 37°C for 1 h. Simultaneously, the pQE-31 expression vector (Qiagen, Germany) was digested with the same enzymes and then purified using the FavorPrep GEL/PCR purification mini kit (Favorgen Biotech Corporation) through gel extraction. The digested insert and vector were ligated overnight at 16°C using T4 DNA ligase (New England Biolabs, USA).

The recombinant plasmid (pQE-31-Cap) was introduced into chemically competent *E. coli* strain M15 cells through heat shock at 42°C for 90 s, followed by recovery in Luria–Bertani (LB) broth (Hardt Diagnostics, USA) at 37°C for 1 h with shaking. Subsequently, transformed cells were plated on LB agar (Titan Biotech Ltd., India) supplemented with 100 μg/mL ampicillin and 25 µg/mL kanamycin (Biobasic Inc., Canada) and incubated at 37°C overnight. Colonies were screened for successful insertion of the *cap* gene through colony PCR using the same primers. Positive clones were validated using Sanger sequencing (Macrogen, Korea) to confirm sequence fidelity and orientation.

For protein expression, a single verified clone was cultured overnight in LB broth supplemented with the appropriate antibiotics, followed by a subculturing process at a 1:50 dilution into fresh LB broth. The culture was incubated at 37°C until the optical density at 600 nm (OD_600_) reached between 0.5 and 0.6. The recombinant Cap protein expression was induced by the addition of 0.5 mM isopropyl β-D-1-thiogalactopyranoside (IPTG), followed by a subsequent incubation at 37°C for 4 h. Cells were harvested by centrifugation at 12,000 × *g* for 10 min at 4°C and subsequently stored at −20°C for future protein purification.

### Protein purification and Western blotting analysis

Cell pellets containing induced *E. coli* M15 cultures were resuspended in phosphate-buffered saline (PBS; pH 7.4) buffer with the addition of 1 mg/mL lysozyme and incubated at 37°C for 60 min. After lysis, the suspension was sonicated on ice using a probe sonicator (OMMI International, USA) set to 30% amplitude, with 10-s pulses, followed by 20-s rests, for a total of 3 cycles. The lysate was then centrifuged at 12,000 × *g* for 15 min at 4°C to isolate the soluble and insoluble protein fractions.

The recombinant Cap protein was found mainly in the insoluble fraction and was solubilized using gradually increasing concentrations of urea (1–8 M) in PBS. The solubilized inclusion bodies were purified under denaturing conditions with NI^2^-nitrilotriacetic acid (Ni-NTA) affinity chromatography using an ÄKTA Start system (GE Healthcare, USA). The column was washed with a buffer containing 8 M urea and increasing concentrations of imidazole (20–60 mM), and the bound protein was eluted with 250 mM imidazole in the same buffer. Recombinant Cap antigen was produced as a single large-scale batch, aliquoted, and stored at −20°C. Each aliquot was used only once to avoid repeated freeze–thaw cycles.

Sodium dodecyl sulfate-polyacrylamide gel electrophoresis (SDS-PAGE) was used to analyze the protein fractions on a 15% polyacrylamide gel under reducing conditions. The gels were stained with Coomassie Brilliant Blue R-250 (Panreac, Spain) to confirm the presence of the target protein at approximately 27 kDa. Proteins were transferred onto a nitrocellulose membrane using a semi-dry blotting system for immunoblotting. Membranes were blocked with 5% non-fat dry milk and 10% normal horse serum in PBS for 30 min at 37°C. Primary detection was performed using either a mouse anti-His tag monoclonal antibody (1:3,000 dilution) (Thermo Fisher Scientific) or DuCV-positive duck serum (1:200 dilution), both of which were incubated at 37°C for 1 h. After three washes with PBS containing 0.05% Tween-20 (PBS-T), the membranes were incubated with 1:1,000 horseradish peroxidase (HRP)-conjugated goat anti-mouse IgG (SeraCare, USA) (for anti-His detection) or goat anti-duck IgG (SeraCare) for 30 min at 37°C. Signal development was immediately visualized using 3,3′,5,5′-tetramethylbenzidine (TMB) membrane substrate (SeraCare).

### Development and optimization of the iELISA

An iELISA was developed using the purified recombinant DuCV Cap protein as the coating antigen. Optimal antigen concentration and serum dilution conditions were determined through checkerboard titration. Ninety-six–well microtiter plates (MaxiSorp, Thermo Fisher Scientific) were coated overnight at 4°C with 100 μL/well of recombinant Cap protein diluted in carbonate–bicarbonate buffer (0.05 M, pH 9.6) at various concentrations (1.5–12 μg/well). After coating, the plates were washed 5 times with PBS-T. They were then blocked at 37°C for 30 min with 300 μL of blocking buffer, composed of 1.5% bovine serum albumin (BSA), 5% non-fat dry milk, 1% casein, 10% normal horse serum, and 10% rabbit serum in PBS, to minimize non-specific background.

Duck sera were diluted in dilution buffer (1:10–1:320) and added to the plates (100 μL/well), followed by incubation at 37°C for 30 min. After washing, 100 μL of HRP-conjugated goat anti-duck IgG (SeraCare) diluted 1:5,000 in PBS was added to each well. Plates were incubated for an additional 30 min at 37°C and then washed 5 times with PBS-T. Color development was achieved by adding 100 μL of SureBlue TMB 1-Component Microwell Peroxidase Substrate (SeraCare) and allowing the reaction to proceed for 15 min at 22°C. The reaction was stopped by adding 100 μL of 0.25 M H_2_SO_4_, and the OD was measured at 450 nm using a microplate reader (Tecan, Switzerland). The optimal conditions were determined based on the highest positive-to-negative (P/N) OD_450_ ratio and minimal background in negative sera. All assays were performed in duplicate.

### Evaluation of assay performance

To determine the cut-off value for iELISA, 103 archived negative serum samples were used. The cut-off value was calculated as the mean OD_450_ of negative samples plus 1, 2, or 3 standard deviations (SD), and the value yielding the best discrimination was chosen for subsequent analyses. The cut-off value was calculated as follows: Assay sensitivity was assessed using 80 serum samples collected from 1-year-old ducks in areas endemic to DuCV that exhibited clinical signs and were confirmed to have infections by PCR. Specificity was evaluated using the 103 confirmed-negative sera described above. Cross-reactivity was assessed using duck sera positive for duck Tembusu virus (DTMUV), duck viral enteritis virus (DVEV), and *Riemerella anatipestifer* from farms with no history or evidence of DuCV infection.

Assay repeatability (intra-assay variation) was evaluated using three reference-positive and three reference-negative duck sera, each tested in 16 replicates on a single plate. Reproducibility across days (inter-assay) was assessed by testing the same sera on three separate days. The % coefficient of variation (CV) for each serum was calculated as 100 × SD/mean within-run and across days. Plate acceptance required the positive control to fall within the laboratory range and the negative control to remain below the cut-off. A single large-scale antigen lot was used for all validation runs and aliquoted to prevent freeze–thaw.

### Comparison with Western blot analysis

A subset of serum samples was tested using both methods to evaluate the diagnostic concordance between the developed iELISA and Western blot analysis. First, the optimal duck serum dilution for use as the primary antibody in Western blotting was determined by serial dilution of pooled DuCV-positive sera (1:10–1:80). The dilution yielding the strongest specific signal with a minimal background was selected and applied to subsequent tests.

A total of 189 individual duck serum samples, obtained from the existing stock collection in our laboratory, were analyzed in parallel by iELISA and Western blot. For Western blotting, purified recombinant Cap protein was separated by SDS-PAGE and transferred onto nitrocellulose membranes as described earlier. The membranes were incubated with duck serum at the optimized dilution, followed by HRP-conjugated goat anti-duck IgG (SeraCare) at a 1:1,000 dilution. Antigen–antibody interaction was visualized using a TMB membrane substrate. An independent observer who was blinded to the ELISA results performed the final interpretation of the Western blot results to minimize observer bias.

Samples were classified as positive or negative based on the presence or absence of specific bands in the Western blot and the corresponding OD_450_ values relative to the established iELISA cut-off. The overall concordance rate and the percentages of positive and negative agreement were used to evaluate the diagnostic agreement between the two methods.

### Statistical analysis

The diagnostic performance of the developed ELISA was evaluated using ROC curve analysis. OD_450_ values from 80 positive and 103 negative sera were analyzed with GraphPad Prism version 10.6.0 (GraphPad Software, Boston, MA, USA). The ROC curve was generated by plotting sensitivity against 1–specificity at various cut-off points. The area under the curve (AUC) was calculated to assess overall accuracy. Furthermore, the diagnostic agreement between the ELISA and Western blot was determined using Cohen’s Kappa coefficient.

## RESULTS

### Confirmation of DuCV infection by PCR

DuCV infection was confirmed in clinical samples using conventional PCR targeting the *rep* gene. As expected, two spleen samples obtained from ducks exhibiting feather abnormalities, emaciation, and growth retardation produced strong PCR amplification products of approximately 620 bp. Amplification was not observed in the non-template control.

### Amplification of the *cap* gene and expression of recombinant Cap protein

**Figure 1:**
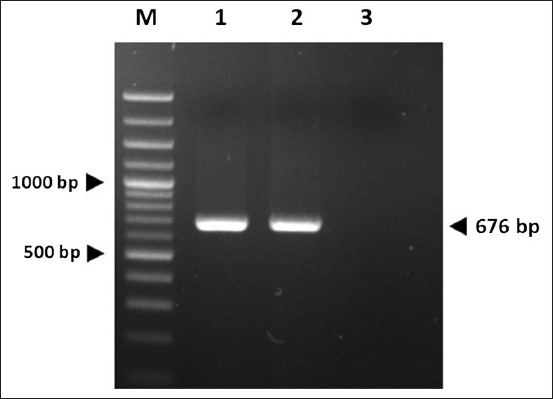
Polymerase chain reaction amplification of the duck circovirus *cap* gene in duck spleen samples using *primers specific to the cap gene* Lane M: DNA marker (DM2400) AccuBand 100 bp+3K DNA Ladder II, Smobio); Lanes 1–2: positive samples (676 bp); Lane 3: non-template control.

**Figure 2:**
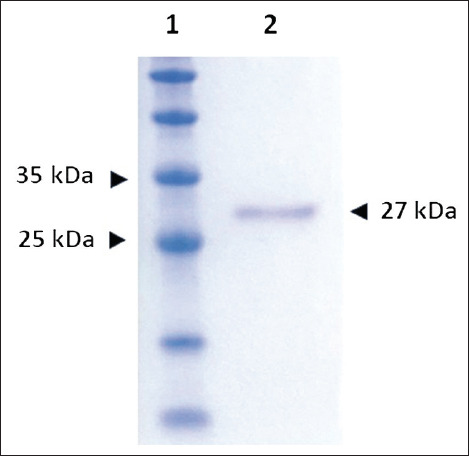
Analysis of recombinant Cap protein by 15% sodium dodecyl sulfate-polyacrylamide gel electrophoresis using (1) a Tricolor Broad Range Retained Protein Ladder (Vivantis, Malaysia) and (2) purified recombinant Cap protein.

The DuCV *cap* gene was successfully amplified using the designed primers DuCV_CAPexF2 and DuCV_CAPexR, resulting in a 676-bp PCR product (Figure 1). Agarose gel electrophoresis confirmed the expected amplicon size, with no non-specific bands. After induction with IPTG, the recombinant DuCV Cap protein was successfully expressed in *E. coli* strain M15. SDS-PAGE analysis revealed a distinct protein band of approximately 27 kDa, consistent with the expected molecular weight of the recombinant Cap protein (Figure 2). The protein was primarily localized in the insoluble fraction. Solubilization using 8 M urea facilitated efficient extraction of the inclusion bodies, and Ni-NTA affinity chromatography was used to purify the recombinant protein under denaturing conditions.

### Immunoreactivity of recombinant Cap protein

**Figure 3:**
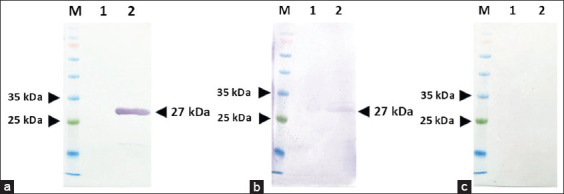
Evaluation of biological Cap protein function by Western blotting. (a) Anti-His tag antibody. (b) Duck circovirus (DuCV)-positive. (c) DuCV-negative duck serum. Lane M: Tricolor Broad Range Retained Protein Ladder (Vivantis, Malaysia); Lane 1: *Escherichia coli* protein; Lane 2: Recombinant Cap protein.

The immunoreactivity of the purified recombinant Cap protein was assessed using western blot analysis. A distinct band at approximately 27 kDa was observed when the protein was bound with a monoclonal anti-His tag antibody, confirming the His-tagged protein’s expression and integrity (Figure 3a). The recombinant protein also produced an immunoreactive signal when tested with DuCV-positive duck serum (Figure 3b), indicating the retention of antigenic epitopes relevant to natural infection. No suspected protein bands were detected when DuCV-negative duck serum was used as the primary antibody (Figure 3c), confirming the specificity of the antigen–antibody interaction. These results validated the use of the recombinant Cap protein as a suitable antigen for developing an iELISA.

### Optimization of iELISA conditions

Optimal conditions for the iELISA were established through checkerboard titration using serial dilutions of the recombinant Cap antigen and duck sera. The highest P/N OD_450_ ratio was achieved when microtiter plates were coated with 12 μg/well of the recombinant protein and duck sera were diluted to 1:20. Under these conditions, the mean OD_450_ for DuCV-positive serum was 0.473, while the mean for DuCV-negative serum was 0.074, resulting in a P/N ratio of 6.4. This configuration was selected for subsequent assay validation due to its high discrimination ability between positive and negative samples and low background noise.

### Diagnostic performance and ROC analysis

**Figure 4:**
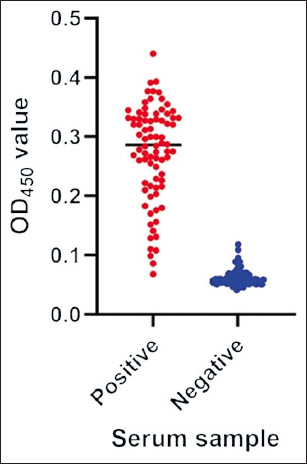
Scatter plots showing the distribution of enzyme-linked immunosorbent assay optical density 450 values of positive (red) and negative (blue) duck serum samples.

The ELISA OD_450_ values of the duck sera are shown in Figure 4. Positive sera (n = 80) yielded consistently higher OD values than negative sera (n = 103), demonstrating a clear separation between the two groups. A total of 103 DuCV-negative serum samples were used to determine the diagnostic cut-off value. The mean OD_450_ for these negatives was 0.061, with a SD of 0.013. Applying the mean + 3SD criterion, the cut-off value was determined to be 0.100.

**Figure 5:**
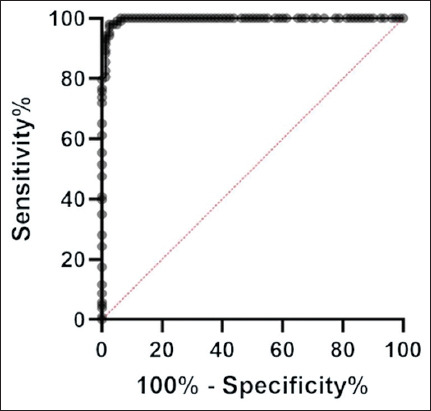
Analysis of the receiver operating characteristic curve of the indirect enzyme-linked immunosorbent assay for detection of duck circovirus antibodies. The area under the curve was 0.996 (95% confidence interval: 0.9906–1.000 with p < 0.001), indicating high diagnostic accuracy of the assay.

Diagnostic performance was further assessed by ROC analysis, yielding an AUC of 0.996 (95% CI, 0.9906–1.000; p < 0.001), indicating excellent discrimination (Figure 5). The ROC-derived cut-off (0.097) closely aligned with the mean + 3SD threshold, confirming the robustness of the initial method. The assay achieved a sensitivity of 97.5% (78/80; 95% CI, 91.34%–99.56%) and a specificity of 98.1% (101/103; 95% CI, 93.19%–99.65%) at this threshold. No cross-reactivity was observed with sera from ducks infected with DTMUV, DVEV, and *R. anatipestifer*, all of which had OD_450_ values below the cut-off.

### Repeatability and reproducibility

Intra-assay evaluation, performed using 16 replicates of three positive and three negative sera, showed %CV values ranging from 2.91% to 6.41% (mean: 4.3%). Inter-assay reproducibility across three independent runs yielded %CV values ranging from 5.87% to 9.09% (mean: 7.1%) (Table 1), confirming the robustness and consistency of the developed iELISA.

**Table 1:** Repeatability and reproducibility of the Cap protein for the iELISA developed in-house, based on sixteen replications for intra-assay and triplicate for inter-assay, using three positive and three negative duck sera. The results are presented as the mean of OD, SD, and %CV.

Samples	Intra-assay			Inter-assay		
	
	Mean OD	SD	%CV	Mean OD	SD	%CV
No. 1	0.226	0.012	5.40	0.211	0.019	9.09
No. 2	0.286	0.011	3.91	0.294	0.019	6.53
No. 3	0.328	0.021	6.41	0.307	0.027	8.89
No. 4	0.065	0.003	4.00	0.063	0.004	5.87
No. 5	0.072	0.002	2.91	0.074	0.005	6.23
No. 6	0.080	0.003	3.16	0.084	0.005	6.01

OD = Optical density, SD = Standard deviation, CV = Coefficient of variation, iELISA = Indirect enzyme-linked immunosorbent assay.

### Comparison of iELISA and Western blotting

To assess diagnostic agreement between the developed iELISA and Western blot, 189 individual duck serum samples were tested using both methods. The optimal serum dilution for Western blot detection was determined to be 1:20 based on signal clarity and background suppression. Of the 189 samples, 172 (91.0%) showed concordant results between the two assays. The positive agreement rate was 94.7% (36/38), whereas the negative agreement rate was 90.1% (136/151) (Table 2). Fifteen samples tested positive by iELISA but negative by Western blot, and two samples were positive by Western blot but fell below the iELISA cut-off. This comparison yielded a Cohen’s kappa value of 0.752 (standard error [SE]= 0.056; 95% CI, 0.642–0.862), indicating substantial agreement between the two methods.

**Table 2:** Comparison of the results of iELISA and Western blot analysis in 189 duck sera.

iELISA	Western blot		Total

	Positive	Negative	
Positive	36	15	51
Negative	2	136	138
Total	38	151	189
% coincidence	94.7	90.1	91.0

iELISA = Indirect enzyme-linked immunosorbent assay.

These discrepancies may reflect differences in assay sensitivity or antibody titers among individual samples. The high level of agreement reinforces the reliability of the iELISA for the routine serological screening of DuCV infection.

## DISCUSSION

### Emergence of DuCV in Thailand and limitations of molecular diagnostics

In 2022, the DuCV genotype I was first reported in circulation among duck farms in Thailand using PCR and sequencing [15]. Although molecular diagnostics, such as PCR and real-time PCR, are highly sensitive and specific for detecting DuCV DNA, these methods are limited to identifying active infections during viral replication [11, 25, 26]. Furthermore, these methodologies do not provide information regarding historical exposure or immune status and may result in false-negative outcomes in patients with intermittent or low-level viremia.

### Development and validation of a recombinant Cap protein-based iELISA

This study developed and validated an iELISA based on a recombinant Cap protein expressed in *E. coli* to detect antibodies against DuCV. The developed iELISA demonstrated excellent diagnostic performance, achieving a sensitivity of 97.5% and a specificity of 98.1%, along with high repeatability and reproducibility. ELISAs can detect antibodies produced by the host, which helps identify both current and past infections, even after viral clearance has occurred. This feature makes ELISA particularly beneficial for large-scale surveillance, epidemiological studies, and flock-level exposure assessment.

Although several DuCV ELISAs have been reported [19, 20, 31], no commercial kits are currently available for antibody detection. This study presents an iELISA using a recombinant Cap protein derived from a Thai genotype I field strain. Unlike prior ELISAs based on subgenotype 2C strains (e.g., GH01 and FJ0601), the antigen used here reflects the circulating subtypes in Thailand, improving assay relevance [15, 19, 20, 31]. A recombinant protein expressed from a sub-genotype that differs from the sub-genotype circulating in another country may affect the sensitivity and specificity of ELISA.

It was previously reported that the amino acid sequences of sub-genotypes (Ib) based on the Cap protein isolated from South Korea exhibited only 84.9%–88.4% identity with sub-genotype 2C strains [6], while genotype III showed <47% identity with genotypes I and II [23]. These differences underscore the importance of selecting locally relevant strains for the design of antigens to ensure diagnostic precision.

### Rationale for antigen selection and protein engineering strategies

The Cap protein was selected for antigen design because of its role as the primary structural and immunogenic component of DuCV [19, 20]. Thirty-six amino acids at the N-terminus of the Cap protein have been identified as the nuclear localization signal (NLS) [33]. Additionally, the region contains numerous arginine-rich sequences. Some residues, known as rare codons, are translated from AGG and AGA codons, which can reduce heterologous protein expression in *E. coli* [34, 35]. The N-terminal region, which contains the NLS and rare codon clusters, was truncated to optimize recombinant expression [19, 20].

In addition, the recombinant Cap protein produced in specific strains of *E. coli* was developed to enhance the expression of eukaryotic proteins, particularly those with rare codons [20, 31]. The full-length *cap* gene, with optimized codons in its NLS regions, has also been expressed in *E. coli* [31]. Codon optimization strategies, including truncation and sequence engineering, significantly enhance protein yield and solubility in prokaryotic systems [31, 36, 37].

In this study, a recombinant Cap protein was produced, excluding the first 26 amino acids, to yield a recombinant Cap protein that retains relevant immunogenic domains, including five common epitopes (A–E) [38]. The developed ELISA demonstrated strong performance in differentiating between positive and negative sera, with a high signal-to-noise ratio and consistent reproducibility across intra- and inter-assay replicates. These results highlight the assay’s robustness and applicability.

### Specificity and cross-reactivity evaluation

The lack of cross-reactivity with sera from ducks infected with DVEV, *R. anatipestifer*, and DTMUV further supports its specificity. Cross-reactivity testing with various pathogens, including *Salmonella* spp., chicken adenovirus serotype 4, Newcastle disease virus (NDV), avian influenza virus (AIV), goose circovirus, and *Pasteurella multocida*, was not performed in this study. Furthermore, since the assay was created using the genotype I antigen, cross-genotype performance against DuCV II and III has not been assessed in this study.

Future studies should involve a larger panel of related pathogens to more thoroughly evaluate possible cross-reactions. However, the overall specificity of the reference-negative panel was strong, and no heterologous reaction was found among the tests.

### Comparison with other diagnostic platforms

A reliable cell culture system for DuCV propagation remains lacking despite attempts to use macrophage-related cells [39, 40]. Serum neutralization testing is currently unfeasible for detecting antibodies against DuCV. In addition to the history of disease outbreaks on the farm, pathogen detection using molecular techniques and a Western blot test was used to confirm the status of positive and negative serum samples [31]. Moreover, both PCR (with a total coincidence rate of 94.6%–95.6%) and Western blot test (with a total coincidence rate of 95.2%) were used to compare the results with those of iELISA [7, 20, 31].

The diagnostic performance of iELISA was further validated through direct comparison with Western blotting, which is widely regarded as a reference method for detecting specific antibody responses. The iELISA achieved a high concordance rate of 91.0% (positive and negative agreement rates of 94.7% (36/38) and 90.1% (136/151), respectively) with Western blotting across 189 individual duck serum samples. This iELISA demonstrates strong diagnostic performance, making it a promising candidate for further development into a standardized commercial kit.

### Assay concordance and diagnostic reliability

These results reflect the strong diagnostic alignment of iELISA with Western blotting in identifying both seropositive and seronegative samples. However, discrepancies were observed in 17 serum samples: 15 sera tested positive by iELISA but were negative by Western blotting, and 2 sera were positive by Western blotting but fell below the iELISA cut-off. These differences likely stem from inherent variations in assay formats and sensitivity thresholds.

Due to its qualitative nature and direct visualization of antigen–antibody binding, Western blotting can detect low-titer or weakly reactive antibodies that produce signals below the threshold of iELISA. These results indicate that the developed assay is a practical and effective tool for sero-surveillance and epidemiological studies of commercial duck populations.

### Applications and future perspectives

Beyond these immediate applications, the assay also provides a foundation for flock-level serological surveys, retrospective testing of archived sera, and the integration of serological data with production records to better understand disease dynamics. It can also be used to monitor antibody responses following vaccination, thereby providing valuable information for evaluating vaccine efficacy and guiding immunization programs.

Such applications will enhance outbreak preparedness, strengthen biosecurity programs, and support the sustainability and competitiveness of duck production in both domestic and international trade.

## CONCLUSION

This study successfully developed and validated an iELISA based on a recombinant Cap protein derived from a Thai DuCV genotype I strain. The recombinant Cap antigen was efficiently expressed in *E. coli*, purified under denaturing conditions, and confirmed by Western blotting to retain immunoreactive epitopes specific to DuCV. The developed iELISA demonstrated excellent diagnostic performance, with a sensitivity of 97.5% and specificity of 98.1%, as well as high intra- and inter-assay reproducibility (%CV < 10%). ROC curve analysis yielded an AUC of 0.996, indicating outstanding diagnostic accuracy. The assay showed no cross-reactivity with sera positive for *R. anatipestifer*, DTMUV, or DVEV, confirming its analytical specificity. Comparative testing with Western blotting produced a substantial diagnostic concordance (Cohen’s kappa = 0.752; overall agreement = 91.0%), underscoring its reliability for serological screening.

The principal strength of this study lies in the use of a recombinant Cap antigen derived from a locally circulating genotype I strain, ensuring region-specific antigenic relevance and improved diagnostic precision. The assay’s robustness, reproducibility, and adaptability make it a practical tool for high-throughput surveillance and epidemiological investigations in commercial duck populations. Moreover, its ability to detect antibodies against both current and past infections enables comprehensive monitoring of flock exposure and immune status.

However, this study was limited by the absence of cross-reactivity testing with a broader panel of avian pathogens (e.g., *Salmonella* spp., AIV, NDV, *P. multocida*) and by its focus on genotype I antigen, leaving cross-genotype reactivity (DuCV II and III) to be evaluated in future work. Additionally, the lack of a cell culture system or neutralization assay for DuCV restricts comparative serological standardization.

In conclusion, the recombinant Cap-based iELISA developed herein represents a sensitive, specific, and reproducible diagnostic platform suitable for large-scale sero-surveillance of DuCV. Its implementation will strengthen diagnostic capacity, enhance biosecurity, and support evidence-based management strategies in the duck industry, contributing to improved flock health and sustainable poultry production.

## AUTHORS’ CONTRIBUTIONS

PL and SK: Designed the research concept and methodology. TL, SJ, and SP: Laboratory work. SK and TL: Collected samples. TL: Analyzed the data and wrote the first draft of the manuscript. PL, SK, SJ, and SP: Reviewed and edited the manuscript. All authors have read and approved the final version of the manuscript.
